# The Administration of Gaseous Enemata for Pulmonary Phthisis

**Published:** 1887-05

**Authors:** 


### CULLINGS FROM CONTEMPORARIES.

### THE ADMINISTRATION OF GASEOUS ENEMATA FOR PULMONARY PHTHISIS.

A large share of professional and public attention has quite re-recently been directed to the administration of gaseous enemata for the treatment of blood poisoning, and of affections of the respiratory passages. The object in view is to supply to the venous circulation an antiseptic, such as hydrogen sulphide, in sufficient doses to be effective; a result impossible when supplied directly to the arterial current, a plan which would poison the patient. Hydrogen sulphide inhaled in far less than sufficient doses would suffocate the patient; taken by the stomach, it would produce other serious results. Administered by the bowels. however, and entering the venous current already deteriorated by organic refuse, it is quickly eliminated by the respiratory tract, which thus becomes subjected to its beneficial local antiseptic effects without subjecting the system at large to injury, as when thrown into the arterial current. In other words, the parasite is killed, without killing the individual. Its beneficial effects in phthisis are explained by the action of the gas on the suppurative and septic surfaces, and not by any influence on the bacillus tuburculosis; the consumption proper, the exhaustion, being due to the suppuration and to the consequent septicæmia, and not immediately to the bacillus, which, while it produces the destruction of tissue, does not produce the morbid phenomena. The method of administration utilizes the discovery announced by Bernard in 1857, that toxic materials introduced into the economy through an organ at a distance from the arterial system could not penetrate into the arterial system because it is eliminated before that system can be reached. Volatile substances are eliminated by thé pulmonary alveoli.

On July 12, 1886, Dr. Bergeon communicated to the French Academy of Sciences the results of several years' investigation into the method, and Professor Cornil also presented later a paper on the subject. The forms of apparatus at present in use are based upon designs furnished by Dr. V. Morel, of Lyons.

Various antiseptic gases and vapors have been tried, but abandoned on account of local irritant action, but a mixture of carbon dioxide (carbonic acid gas) and hydrogen sulphide (sulphuretted hydrogen) is entirely harmless when properly used and completely deprived of atmospheric air.

Since the object of this article is entirely practical, it will not be necessary to discuss the physiological action or the therepeutical theories involved. There will simply be presented descriptions of the more recent forms of the apparatus and of the method of use.

The apparatus exhibited in the accompanying cuts was designed by Mr. J. A. Kyner, superintendent of the Polyclinic, in imitation of one of Morel's apparatus lately imported by Dr. J. Solis-Cohen.

The apparatus as constructed by Mr. Kyner is now in use at the hospital of the University of Pennsylvania, German Hospital, Home for Consumptives, and by quite a number of private practioners, and the form is now manufactured by Messrs. James W. Queen & Co., of this city.

The apparatus consists of a generator, a reservoir, a bulb apparatus for injection, and a vessel for holding the sulphur water. To generate the carbon dioxide, put one avoirdupois ounce of sodium bicarbonate and one fluid ounce of water into the wide-mouth jar; close the jar with the rubber stopper carrying the funnel-tube and short delivery-tube. Fill the funnel with dilute sulphuric acid, made by adding four fluid drachms of strong acid to four fluid drachms of water. By means of the stop-cock on the funnel-tube, allow about a teaspoonful of the acid to run into the bottle so as to generate sufficient gas to expel the air in the bottle. Then, having rolled the reservoir tightly to exclude all air, connect it by means of the rubber hose to the generator, and continue the slow addition of the acid from the funnel-tube until the reservoir is filled. The quantities above given for charging the generator will be found about sufficient to fill the reservoir. Dr. Bergeoin recommends that the acid be prepared at the bedside, but Mr. Kyner has used it entirely successtully after being kept six hours in a heavy vulcanized rubber bag, such as is now furnished; but Dr. Bergeoin used a lighter bag, which had not the power of resisting diffusion. This probably explains the difference in results. When the reservoir is filled it is detached from the hose, and the stopcock immediately closed.

To administer the gas, the reservoir is attached to the free end of the syringe bulb; the wash-bottle being about three-fourths filled with sulphur water is stood in a basin of warm water and closed by the rubber stopper carrying two tubes, attached to the other end of the syringe bulb. The stopcock of the reservoir is opened and sufficient gas forced through by means of the syring bulb to expel the air from the wash-bottle and tubes; the hard-rubber vaginal syringe pipe is then well inserted into the rectum, and the gas pumped very slowly. From one to six quarts is the amount administered according to circumstances. From four to five minutes should be allowed for the injection of each quart. The patient should lie on the right side or on the back. Should any difficulty occur from the escape of gas from the rectum, the patient's legs should be extended so as to compress the sphincter.

It is the universal statement of patients that the injection can be given more satisfactorily and with less uneasiness when the bowels have been emptied. Two injections a day should be gived. Since the injection interferes slightly with digestion, it should be given either one hour before or three hours after a meal. No pain except that of slight distention of the bowels is felt unless air is present in the apparatus. The natural sulphur waters used in this city are the Red Sulphur Springs, of Virginia, and the Mount Clemmens water of Michigan; the latter is to be preferred since it contains about ten times the available sulphur compounds. The same portion has been used satisfactorily for three consecutive injections, and still smelled strongly of the gas. Although artificial waters have been said to cause pain, the following formulæ have been used without any difference of effect from natural waters having been noticed by the patient:

| | | | | |
|---|---|---|---|---|
| ℞. | Sodium sulphide, pure. | | | |
| | Sodium chloride, | aa | gr. v | |
| | Water, | | f℥xxij. | M. |

This is the formula first used at the Philadelphia Hospital. The hydrogen sulphide is formed by the action of the carbonic acid on the sodium sulphide substantially according to the following reaction:—

$$Na_2S \text{ plus } H_2CO_3 = Na_2CO_3 \text{ plus } H_2S.$$

When pure sodium sulphide is not attainable, the *potassium sulphuretum* or corresponding sodium compound may be used. These must be used in rather larger proportion, and produce an objectionable white precipitate of sulphur.

When a stronger sulphur water is desired than that produced by the above formula, the following may be used:—

| | | |
|---|---|---|
| ℞. | Sodium sulphide, pure, | gr. x |
| | Dilute hydrochloric acid, U. S. P., | mxxx |
| | Water, | f℥xxii. |

Mr. Kyner, who has proposed this formula, prefers to keep the liquid on hand after use, and freshen it up for subsequent use by additional quantities of sodium sulphide and dilute hydrochloric acid. This freshening up should be done whenever the liquid ceases to smell of the hydrogen sulphide. A liquid so kept seems to acquire more nearly the characteristic odor of the natural water. If the sulphur water is of sufficient strength, the patient's breath will, in about five minutes after beginning the administration, darken lead acetate paper, and will continue to smell of gas for an hour after the process is discontinued. It may be well to remark that metals, especially silver, are readily tarnished by the sulphur gases.

The method has, up to the present, been used upon about one hundred cases in this city without any untoward effects, so far as known, except in one or two instances, one of which was due to a leaky bag, and another to incorrect administration.

It is, perhaps, too soon to decide positively on the therapeutic value of the new method, but it seems in the experience in this city to have the special quality of diminishing night sweats and improving the appetite.

In Bergeon's cases, the trifling expectorations of those apparently practically cured continued to contain bacilli. This fact may be taken both for an indication that the immediate danger in phthisis is less from the bacilli than from the septicæmia which they set up, and as an indication that this protective treatment, when successful, should not be discontinued until the general healthiness of the tissues is sufficiently restored to resist the further development and sustenance of the bacillus tuberculosis.—*The Polyclinic.*

